# The Impact of FFC-AC-EIT Upon Patient Outcomes

**DOI:** 10.1093/geroni/igaf122.723

**Published:** 2025-12-31

**Authors:** Barbara Resnick, Boltz Marie, Ashley Kuzmik

**Affiliations:** University of Maryland, Baltimore, Maryland, United States; The Pennsylvania State University, University Park, Pennsylvania, United States; Alzheimer’s Association, Chicago, Illinois, United States

## Abstract

This study tested the impact of FFC-AC-EIT on hospitalized patients living with dementia. A total of 455 patients were recruited from 12 hospitals. The mean age of the participants was 82.5 (SD = 8.5) and the majority were women (62.6%), White older adults (65.3%), married (64.4%) and with high school or less education (67%). All had evidence of cognitive impairment with a mean SLUMS score of 7.5 (SD = 5.9) and a mean of 3.0 (SD = 2.4) comorbidities. At discharge, both FFC-AC-EIT and Education-only groups improved in function and physical activity, patients in the FFC-AC-EIT group had less increase in pain than those in the Education-only group (β = -0.58, [95% CI] (-0.89, -0.26), p < 0.001), with no group differences in behavioral symptoms or delirium. At one month post-discharge there was a significant improvement in function (β = 5.55, 95% CI [0.36, 10.75], p = 0.036) and physical activity (β = 3.07, 95% CI [1.87, 4.28], p < 0.001) in the FFC-AC-EIT arm. The decrease in pain between admission and discharge indicates that addressing pain and engaging patients in FFC-AC-EIT did not result in exacerbation of pain. Exposure to information about FFC-AC-EIT, including Education-only, may help hospital staff maintain and increase function and physical activity for older patients living with dementia and help facilitate an increase in function and physical activity after discharge.

